# Neuroanatomical correlates of aggressiveness: a case–control voxel- and surface-based morphometric study

**DOI:** 10.1007/s00429-023-02715-x

**Published:** 2023-10-11

**Authors:** Stephanie Seidenbecher, Maria Schöne, Jörn Kaufmann, Kolja Schiltz, Bernhard Bogerts, Thomas Frodl

**Affiliations:** 1 Department of Psychiatry and Psychotherapy, Otto von Guericke University Magdeburg, Leipziger Str. 44, 39120 Magdeburg, Germany; 2 Department of Neurology, Otto von Guericke University Magdeburg, Magdeburg, Germany; 3 Department of Forensic Psychiatry, Psychiatric Hospital of the Ludwig-Maximilians-University, Munich, Germany; 4 Center for Behavioral Brain Sciences (CBBS), Otto von Guericke University Magdeburg, Magdeburg, Germany; 5 Salus-Institute, Salus gGmbH, Magdeburg, Germany; 6 Department of Psychiatry and Psychotherapy, RWTH Aachen University, Aachen, Germany

**Keywords:** Aggression, Voxel-based morphometry (VBM), Surface-based morphometry (SBM), Magnetic resonance imaging (MRI), Martial arts

## Abstract

Aggression occurs across the population ranging on a symptom continuum. Most previous studies have used magnetic resonance imaging in clinical/forensic samples, which is associated with several confounding factors. The present study examined structural brain characteristics in two healthy samples differing only in their propensity for aggressive behavior. Voxel- and surface-based morphometry (SBM) analyses were performed on 29 male martial artists and 32 age-matched male controls. Martial artists had significantly increased mean gray matter volume in two frontal (left superior frontal gyrus and bilateral anterior cingulate cortex) and one parietal (bilateral posterior cingulate gyrus and precuneus) brain clusters compared to controls (whole brain: *p* < 0.001, cluster level: family-wise error (FWE)-corrected). SBM analyses revealed a trend for greater gyrification indices in martial artists compared to controls in the left lateral orbital frontal cortex and the left pars orbitalis (whole brain: *p* < 0.001, cluster level: FWE-corrected). The results indicate brain structural differences between martial artists and controls in frontal and parietal brain areas critical for emotion processing/inhibition of emotions as well as empathic processes. The present study highlights the importance of studying healthy subjects with a propensity for aggressive behavior in future structural MRI research on aggression.

**Supplementary Information:**

The online version contains supplementary material available at 10.1007/s00429-023-02715-x.

## Introduction

Human aggression is a complex, heterogeneous, and multifactorial construct (Dorfman et al. 2014) with multiple motives and triggers (Wahlund and Kristiansson 2009), and thus has a wide range of different manifestations in the population (Dambacher et al. 2015a; Besteher et al. 2017). It is defined as any type of hostile, injurious, or destructive behavior (Siever 2008) toward other people or living beings that causes them physical or psychological harm (Anderson and Bushman 2002; Perach-Barzilay et al. 2013). The term aggression encompasses different varieties of behaviors, cognitions, and emotions that can range from mild irritability to overtly violent behavior (Rosell and Siever 2015; Blair 2016). In addition to the conventional classification into reactive/impulsive and proactive/instrumental aggression (Flanigan and Russo 2019), Dorfman et al. (2014) distinguish between functional and pathological forms of aggression. Functional aggression is contextually appropriate and constrained by norms and rules (e.g., American football, martial arts), whereas pathological aggression is decontextualized, unstructured, and does not conform to rules (e.g., aggressive symptoms in antisocial personality disorder (APD) or Psychopathy) (Dorfman et al. 2014).

In its extreme form, aggressive behavior can lead to serious crimes with drastic physical and/or emotional consequences for victims and impose enormous costs on society (Dambacher et al. 2015a; Zhang et al. 2019). Therefore, deeper understanding of the correlates of aggressive behavior is crucial for the development of effective interventions for prevention and targeted treatment. In recent years, structural [voxel-based morphometry (VBM), surface-based morphometry (SBM)] and functional (functional magnet resonance imaging (fMRI), resting-state fMRI) neuroimaging techniques have emerged as powerful tools to study the neural basis of aggression (Bufkin and Luttrell 2005; Raschle et al. 2015). In particular, studies of the brain morphology in aggressive populations can provide evidence for causality and offer information about mechanisms that contribute to the maintenance of aggressive behavior (Schiffer et al. 2011). These studies have pointed to morphological differences underlying aggression in several brain structures, particularly the frontal and temporal lobes (Kumari et al. 2014; Bannon et al. 2015; Dambacher et al. 2015a; Peper et al. 2015; Smith et al. 2016), which are involved in emotion processing and behavior regulation (Rosell and Siever 2015; Leutgeb et al. 2016). In particular, the prefrontal cortex (PFC), especially the orbitofrontal cortex (OFC), the medial temporal cortex, the amygdala, the basal ganglia, and the anterior cingulate cortex (ACC) show structural and/or functional changes that appear to be strongly associated with aggressive behavior (Rosell and Siever 2015; Leutgeb et al. 2016).

Most previous studies on aggressive/violent behavior have been conducted with psychiatric patients, e.g., participants with schizophrenia (Hoptman and Antonius 2011; Fjellvang et al. 2018), borderline personality disorder (Bertsch et al. 2013), bipolar disorder (Soloff et al. 2014), APD (Hoptman 2003; Wahlund and Kristiansson 2009), or incarcerated/forensic participants with Psychopathy (Koenigs 2012; Johanson et al. 2020), and youths/adolescents with disruptive behavior disorder (Baker et al. 2015). Despite the extensive database, this approach presents several methodological challenges. A high proportion of clinical or incarcerated/forensic patients have comorbidities with other mental disorders, such as schizophrenia, organic brain syndrome (Schiltz et al. 2013), anxiety disorders, or various personality disorders, and especially substance abuse (Loeber et al. 2000; Palijan et al. 2010; Ermer et al. 2013). Alcohol abuse, for example, is associated with structural changes throughout the whole brain, most pronounced in the frontal and temporal cortex (Fortier et al. 2011). Cannabis abuse can lead to structural changes in the frontal and insular cortex (Lopez-Larson et al. 2011). Therefore, the results of studies examining these populations may be poorly interpreted due to confounding variables that are difficult to exclude. In addition, the selection of well-matched control subjects is difficult and critical. In previous studies, a common control group consisted of non-criminal, mentally healthy individuals (Wahlund and Kristiansson 2009). In addition to comorbid conditions, other important variables, such as age, education, or cognitive ability, may influence brain structure. Therefore, differences between groups may result from these confounding factors (Howner et al. 2012; Koenigs 2012). Another critical issue is the heterogeneity of current studies in terms of inclusion and exclusion criteria. Often, diagnoses are not clearly differentiated: some studies examine patients with psychopathy (Howner et al. 2012), APD (Kumari et al. 2014), or both diagnoses (Bertsch et al. 2013), while others examine violent individuals in general (Hofhansel et al. 2020). Some studies examine convicted offenders (Verdejo-Román et al. 2019), sometimes with recording of psychopathic traits (Ermer et al. 2012), while others examine un-convicted subjects with psychopathic traits (Pardini et al. 2014). Moreover, in several studies, the diagnostic definition of the included individuals is not entirely clear. Therefore, it is difficult to draw general conclusions from many of the current studies due to the unclear group characterizations (Raschle et al. 2015).

In contrast to the aforementioned studies in clinical/forensic samples, which must contend with multiple confounding factors, there is limited structural neuroimaging research in adult, mentally healthy, community-based samples with no history of incarceration that represent the lower end of the symptom continuum of aggression (Garvey et al. 2016; Besteher et al. 2017; Coccaro et al. 2018). In a morphometric study, Matsuo et al. (2009) examined the relationship between gray matter (GM) volume of the ventromedial PFC and impulsivity in healthy participants. The GM volume of the right OFC was inversely correlated with non-planning impulsivity, whereas left OFC GM volume was inversely correlated with motor impulsivity (Matsuo et al. 2009). In another morphometric study, Matthies et al. (2012) examined the relationship between amygdala volume and lifetime aggression in healthy female participants. They report a 16–18% reduction in amygdala volume in participants with higher aggression scores. Amygdala volume is significantly inversely related to trait aggression (Matthies et al. 2012). Besteher et al. (2017) examined brain structural correlates of irritability in mentally healthy adults using VBM and SBM. They report significant positive correlations between GM volume and aggression/hostility in large clusters involving the bilateral ACC and OFC as well as the left lingual and postcentral gyri. For SBM measures, they describe positive correlations of aggression/hostility with cortical thickness in the bilateral precentral gyri and with the gyrification index (GI) in the left insula and superior temporal gyrus (Besteher et al. 2017). Finally, Coccaro et al. (2018) used VBM to investigate the relationship between lifetime aggression and GM volume in healthy adult same-sex twins. They describe an inverse association between medial and lateral PFC GM volume and lifetime aggression (Coccaro et al. 2018). Common to all these studies is that they are purely correlative and thus do not include a well-matched control group with no propensity for aggressive behavior. Moreover, the samples in these studies consist of healthy individuals who are within the normal range of aggression/irritability and thus within the normal range of psychological functioning (Matsuo et al. 2009; Matthies et al. 2012; Besteher et al. 2017).

Whether healthy subjects with a propensity for aggressive behavior differ in their brain structure from control subjects without a history of aggressive behavior has not yet been explored. Therefore, in a first study, we investigated GM concentration differences in 21 male healthy martial artists compared to 26 male healthy controls and their association with aggressiveness using VBM (Breitschuh et al. 2018). We describe an interaction effect between group membership and aggressiveness in a cluster comprising the left temporal pole and the left inferior temporal gyrus. In martial artists, aggressiveness is inversely related to mean GM concentration in these brain regions, whereas the opposite pattern is observed in controls (Breitschuh et al. 2018). However, there were methodological limitations of this initial study that should be noted and require further thorough investigation. First, the experimental group of martial artists was heterogeneous in terms of the type(s) of sport (self-defense vs. full-contact sport) and the combat experience. Important potentially moderating variables were not examined, e.g., physical activity, psychopathic traits, early childhood trauma, etc. Finally, we did not perform surface measurements that have been shown to increase the accuracy of brain registration (Desai et al. 2005).

The present study examines brain morphological differences relevant to aggression in non-clinical/non-forensic samples. An experimental group prone to aggressive behavior is compared to a well-matched healthy comparison group, controlling for several demographic, behavioral, and psychiatric factors that commonly co-vary with aggression. For this purpose, differences in GM volume are examined using whole-brain VBM (Ashburner and Friston 2000; Good et al. 2001). Cortical thickness and GI differences are examined using SBM (Yotter et al. 2011; Dahnke et al. 2013) to investigate finer differences in brain morphology. In this study, we therefore test the hypothesis of a relationship between aggressiveness and structural brain changes in healthy community adults with different propensities for aggressive behavior.

## Methods

### Participants

Between 2017 and 2020, *n* = 33 martial artists (male, age: *M* = 25.52 ± 5.46 years) from local fight clubs and *n* = 38 controls (male, age: *M* = 27.05 ± 6.28 years) from the community were recruited via announcements in the city of Magdeburg, social media posts in local groups, and information on the Otto von Guericke University Magdeburg website. Exclusion criteria were age less than 18 or greater than 50 years, female or diverse gender, left-handedness or ambidexterity, self-reported history of neurological or psychiatric disorders including alcohol/substance dependency, self-reported history of criminal offenses, head injury resulting in unconsciousness, and MRI contraindications. Because adolescence is a period of significant biological, cognitive, and neural change processes (Sweeten et al. 2013), only adults were included. In addition, we studied only male participants because sex hormones modulate brain morphology (Pletzer 2019) and we wanted to avoid this confounding effect of gender. Specifically, the inclusion criteria for martial artists were at least one year of martial arts experience and regular training at the time of measurement. In addition, only ‘hard’ martial arts were included, specifically full-contact sports characterized by kicking and punching techniques and kumite (a type of sparring) (Nosanchuk 1981; Vertonghen et al. 2014), such as Mixed Martial Arts, kickboxing, and Muay Thai. Approximately 30% of the martial artists screened were eventually enrolled in the study. In contrast, control subjects were not expected to have martial arts experience. Three participants (*n* = 2 martial artists) were excluded because they were ambidextrous. Another *n* = 3 participants (*n* = 1 martial artist) were excluded because they were regular drug users. One martial artist was excluded because he did not regularly practice martial arts at the time of measurement. Furthermore, *n* = 1 control subject was excluded because of low martial arts experience (3 months) and *n* = 2 control subjects were excluded because they reported participating in fights related to the local soccer ultra-scene. This results in a final sample of *n* = 29 martial artists and *n* = 32 controls.

All participants gave written informed consent prior to enrollment in the study according to the procedures approved by the institutional review board of the Faculty of Medicine (Otto von Guericke University Magdeburg). They received financial compensation for their participation. The study was conducted in accordance with The Code of Ethics of the World Medical Association (Declaration of Helsinki).

### Measures

The entire study consisted of two measurement days at the Department of Psychiatry and Psychotherapy at Magdeburg University Hospital. On the first day, the aims of the study were explained to the participants and they gave their written informed consent. A 1.5-h 3 Tesla MRI session (including T1-weighted MRI, resting-state fMRI, and fMRI) was performed. Behavioral assessment (ratings, self-report scales) was then performed. On the second day, participants completed a cognitive performance assessment. This was followed by another 1.5-h 3 Tesla MRI session (including T1-weighted MRI, fMRI, and DTI). In addition, the digit ratio ‘index finger’ to ‘ring finger’ (2D:4D ratio) of the right hand was determined, which correlates with the balance of fetal testosterone and estrogen (Manning et al. 2014). Only a subset of assessments and tests were selected for the present statistical analyses, and these are described in more detail in the following sections.

### Handedness

Handedness was assessed using the Edinburgh Handedness Inventory (EHI; Oldfield 1971). This is a ten-item self-report questionnaire designed to determine which hand is preferred for various activities (e.g., writing, drawing, throwing). If the right hand is preferred, there are + 10 points. If the left hand is preferred, there are −10 points, otherwise 0 points. A total score of more than 40 points means that the person is right-handed (Oldfield 1971).

### Psychiatric conspicuousness

The Brief Psychiatric Rating Scale (BPRS; CIPS 1977) assesses a wide range of psychopathological symptoms. This scale consists of 18 items that have to be rated on a seven-point Likert scale ranging from 1—“nonexistent” to 7—“extremely severe”, and records symptoms on five subscales of “anxiety/depression”, “anergia”, “thought disturbance”, “activation” and “hostility”.

The Childhood Trauma Questionnaire (CTQ; Wingenfeld et al. 2010) is a 28-item self-report measure of childhood and adolescent experiences. Each item can be rated on a five-point Likert scale ranging from 1—“not at all” to 5—“very often”. The questionnaire has five subscales: “emotional abuse”, “emotional neglect”, “physical abuse”, “physical neglect”, and “sexual abuse”. In addition, ‘trivialization/denial’ is assessed for control purposes.

The Psychopathic Personality Inventory-Revised (PPI-R; Alpers and Eisenbarth 2008) is a self-report instrument with 154 multiple-choice questions that measure the personality construct Psychopathy. Each item must be rated on a four-point Likert scale ranging from 0—“not true” to 3—“completely true”.

### Intelligence

The Multiple-Choice Vocabulary Intelligence Test (MWT-B; Lehrl 2005) is a measure of premorbid and crystalline intelligence. The intelligence quotient (IQ) reflects the verbal-theoretical intellectual structures of the participant.

The Performance Testing System, subtest 3 (LPS-3; Horn 1983) is a non-verbal test of logical-deductive reasoning. This test is considered as an indicator of fluid, education-independent intelligence.

### Physical activity

The Global Physical Activity Questionnaire (GPAQ; WHO 2005) is a self-report questionnaire that uses 16 multiple-choice or open-ended questions to collect information on participation in physical activity in the three domains: (1) activity at work, (2) travel to and from places, and (3) leisure-time activities. The calculated Metabolic Equivalent (MET) is an indicator of the intensity of the physical activity.

### Aggression

Aggression in the sample was assessed using two different self-report instruments: The Appetitive and Facilitative Aggression Scale [AFAS; work in progress, civilian version of the Appetitive Aggression Scale (Cronbach’s *α* = 0.85, Weierstall and Elbert 2011)] and the Buss–Perry Aggression Questionnaire (BPAQ; Cronbach’s *α* = 0.62–0.82, Werner and von Collani 2014). The AFAS consists of 30 items that ask about reactions to frustrations or injustices over the course of one’s life, which must be rated on a five-point Likert scale ranging from 0—“never” to 4—“very often”, and which can be divided into two subscales, ‘appetitive aggression’ and ‘facilitative aggression’. The BPAQ consists of 29 items rated on a four-point Likert scale ranging from 1—“not applicable” to 4—“completely applicable”. They represent the four subscales “physical aggression” and “verbal aggression”, which display behavioral tendencies in the form of reactive aggression as well as “anger” (affective component) and “hostility” (cognitive component) (Buss and Perry 1992; Werner and von Collani 2014). As mentioned in previous studies (Karlsgodt et al. 2015), we also calculated the two combined scores of “aggressive actions” (physical + verbal aggression) and “aggressive thoughts” (anger + hostility).

### Empathy

Because empathy modulates the individual aggression risk (Blair 2018), we assessed it using the Questionnaire of Cognitive and Affective Empathy (QCAE; Cronbach’s *α* = 0.65–0.85, Reniers et al. 2011). The QCAE measures empathy on two subscales, “cognitive empathy” (including perspective taking and online simulation) and “affective empathy” (including emotion contagion, proximal responsivity and peripheral responsivity). The questionnaire consists of 31 items that must be rated on a four-point Likert scale ranging from 1—“strong rejection” to 4—“strong approval”.

### MRI image acquisition

Structural images were acquired using a 3 Tesla Siemens (MAGNETOM Prisma syngo MR D13D; Siemens, Erlangen, Germany) MRI scanner with a 64-channel phased-array head coil and a magnetization-prepared rapid gradient echo (MPRAGE) sequence. All subjects received earplugs to protect them from noise in the head coil. High-resolution T1-weighted three-dimensional anatomical scans of 192 sagittal slices with a voxel size of 1.0 × 1.0 x 1.0 mm^3^ of the whole brain were obtained (repetition time (TR): 2500 ms, echo time (TE): 2.82 ms, inversion time (TI): 1100 ms, field of view (FOV) of foot to head (FH): 256 mm, anterior to posterior (AP): 256 mm and right to left (RL): 192 mm, flip angle of 7°, matrix size = 256 × 256). The scan time required for structural acquisition was 9 min 20 s.

To exclude any brain abnormalities or pathological changes detectable by qualitative visual inspection of the scans, such as ventricular enlargement, and subcortical or cortical tissue damage, all T1-weighted raw images of the martial artists and controls were arranged in random order and evaluated under blinded conditions by two independent research psychiatrists (B. B., T. F.) trained in neuroanatomical and radiological assessment. No enlargement of the cavum septum pellucidum, which is associated with aggressive behavior (White et al. 2013), or other brain abnormalities were found.

### MRI data analysis

All data were converted to the Neuroimaging Informatics Technology Initiative (NIfTI) format for analysis using MRIcron (Rorden et al. 2007). MRI data were preprocessed and analyzed in MATLAB (version R2021b; The MathWorks, Natick, MA, USA) using the Statistical Parametric Mapping analysis package (SPM12, version: 7771; Wellcome Trust Centre for Neuroimaging, London, UK) and the Computational Anatomy Toolbox for SPM (CAT12, version: CAT12.8.2; Structural Brain Mapping Group, Jena, Germany) for SBM (cortical thickness, GI) and VBM (GM volume) analyses according to standard procedures.

### Voxel-based morphometry

All T1-weighted images were corrected for bias-field in-homogeneities and segmented into GM, white matter, and cerebrospinal fluid (CSF) (Ashburner and Friston 2005). The images were then spatially normalized using the DARTEL algorithm (Ashburner 2007). In addition, partial volume effects were taken into account as part of the segmentation process (Tohka et al. 2004). Besides visual inspection for artifacts, an automated quality control was performed. As a result, *n* = 1 martial artist was excluded based on weighted overall image quality below two standard deviations and *n* = 3 controls were excluded based on Mahalanobis distances or weighted overall image quality below two standard deviations. The remaining sample (*n*_*MA*_ = 28, *n*_*C*_ = 29) had median image quality ratings (IQR) of *Mdn*_*MA*_ = 86.50 (*Q*1 = 86.35, *Q*3 = 86.61) and *Mdn*_*C*_ = 86.47 (*Q*1 = 86.36, *Q*3 = 86.61), both corresponding to level B. Total intracranial volume (TIV) was calculated. The resulting modulated normalized GM images were smoothed with an 8 mm full width at half-maximum (FWHM) Gaussian kernel. To account for artifacts at the GM/WM border (e.g., incorrect voxel classification), an absolute GM threshold of 0.1 was applied.

For whole-brain group analyses, the normalized and smoothed GM images (dependent variable) were entered into a general linear model (two-sample *t*-test in SPM12), where group (martial artists, control subjects) was a factor and age and TIV were added as covariates to remove any associated variance. We examined the two contrasts “contrast 1: controls > martial artists” and “contrast 2: martial artists > controls” using t tests. To account for multiple comparisons, we chose a whole-brain statistical threshold of *p* < 0.001 (uncorrected), followed by a family-wise-error (FWE) correction *p*_*FWE*_ < 0.025 (corrected for the number of t contrasts calculated) at cluster level. The cluster-level correction used is the one implemented in SPM and displayed in its results window. It is based on the Gaussian Random Field Theory (Brett et al. 2003; Worsley et al. 1996). Based on the voxel peak values, different anatomical regions resulting from the between-groups comparisons were labeled according to the CAT12 automated anatomical atlas 3 (AAL3; Rolls et al. 2020) for Diffeomorphic Anatomical Registration using Exponentiated Lie algebra (DARTEL; Ashburner 2007) space.

### Surface-based morphometry

Analogous to the preprocessing steps for VBM analysis, the anatomical images were spatially normalized and segmented. In a next step, indices for cortical thickness (Dahnke et al. 2013) and gyrification (Luders et al. 2006) were calculated. The CAT12 toolbox provides a projection-based thickness estimation that can be used to calculate cortical thickness and the central surface in one step (Dahnke et al. 2013). GI was extracted from the central surface data based on the absolute mean curvature (Luders et al. 2006). Automated quality control was performed, as a result of which no participant had to be excluded. In addition, for surface data, the Euler number and the size of topology defects were added to the quality check. Finally, T1-weighted anatomical images of the left and the right hemispheres were resampled and smoothed with FWHM Gaussian kernels. According to the matched-filter theorem, smoothing with a Gaussian kernel of 15 mm was performed for cortical thickness and 25 mm smoothing was applied for GI.

For the second-level analyses, the resampled and smoothed images of cortical thickness and gyrification (dependent variables) were entered into two general linear models (two-sample *t* tests), where group (martial artists, control subjects) was a factor and age was a covariate of no interest. We performed whole-brain analyses examining the two contrasts “contrast 1: controls > martial artists” and “contrast 2: martial artists > controls” using t tests. To account for multiple comparisons, we chose a whole-brain statistical threshold of *p* < 0.001 (uncorrected), followed by a FWE correction *p*_*FWE*_ < 0.025 (corrected for the number of *t* contrasts calculated) at cluster level. Cluster labeling was performed using the Desikan–Killiany atlas (Desikan et al. 2006).

### Statistical analysis

SPSS Statistics (Version 26.0.0.0; IBM, New York, USA) was used for statistical analyses. All data were tested for normal distribution using Shapiro–Wilk tests. Two-sample *t* tests and Pearson correlations were performed for normally distributed data (*p* > 0.05); otherwise, Mann–Whitney *U* tests and Spearman correlations were performed. Chi-squared or Fisher’s exact tests were used to analyze categorical variables.

For group comparisons, the mean GM volumes of significant clusters were extracted for each subject using the REX toolbox (Gabrieli Lab, Massachusetts Institute of Technology, Cambridge, Massachusetts, USA). Cortical thickness and GI values for significant regions of interest were likewise extracted. All these mean values were entered into SPSS Statistics, and post hoc partial correlations (Pearson partial correlation for normally distributed data or non-parametric partial rank correlation for non-normally distributed data) with the psychometric scales (AFAS, BPAQ, and QCAE) were performed controlling age and TIV. The significance threshold was set at *p* < 0.05.

## Results

### Descriptive data

Martial artists (*M* = 25.59 ± 5.73 years) and controls *(M* = 28.00 ± 6.14 years) did not differ significantly in age (*T*(59) = –1.58, *p* = 0.119), highest level of education (*p* = 0.059), and crystalline (*U* = 426.00, *Z* = −0.55, *p* = 0.582) or fluid (*T*(59) =  −1.04, *p* = 0.301) IQ scores. In addition, there were no significant differences between the two groups in marital status (*p* = 0.633), handedness (*U* = 404.50, *Z* = −0.89, *p* = 0.376), balance of fetal testosterone and estrogen (*T*(59) = 1.75, *p* = 0.085), BMI (*U* = 456.00, *Z* = −0.12, *p* = 0.908), and physical activity (*U* = 371.00, *Z* = −1.34, *p* = 0.179). There was no evidence of psychiatric symptoms, psychopathic traits, or early childhood stress in the present sample (see Supplementary Table S1). All participants reported being physically healthy and never having received psychological or neurological treatment. Six participants reported taking medication at the time of measurement: *n* = 2 antihistamines (*n* = 2 martial artists), *n* = 2 thyroid hormones (iodide, l-thyroxine; *n* = 1 martial artist), *n* = 2 bronchodilators (inhalation powder; *n* = 2 martial artists). Regarding nicotine use, there was no significant association between group membership and smoking, χ^2^(1) = 0.41, *p* = 0.840. Table 1 provides a detailed overview of the sample characteristics (see “Measures” for detailed description).

**Table 1:** Sample characteristics of the martial artists compared to controls

	Martial artists (*n* = 29)	Controls (*n* = 32)	Statistics
Age (years)	*M* = 25.59 ± 5.73	*M* = 28.00 ± 6.14	*T*(59) = −1.58, *p* = 0.119
Highest level of education (number)			*p* = 0.059
Lower secondary school certificate	*n* = 1	*n* = 0	
Secondary school certificate	*n* = 6	*n* = 2	
High-school diploma	*n* = 14	*n* = 12	
Polytechnic/university degree	*n* = 8	*n* = 18	
Marital status (number)			*p* = 0.633
Single	*n* = 11	*n* = 16	
In relationship	*n* = 15	*n* = 14	
Married/registered partnership	*n* = 3	*n* = 2	
Handedness (EHI)	*Mdn* = 90.00 (*Q*1 = 70.00, *Q*3 = 100.00)	*Mdn* = 80.00 (*Q1* = 70.00, *Q3* = 100.00)	*U* = 404.50, *Z* = −0.89, *p* = 0.376
Body-mass-index (BMI) (kg/m^2^)	*Mdn* = 24.49 (*Q*1 = 23.02, *Q*3 = 26.34)	*Mdn* = 24.88 (*Q*1 = 22.09, *Q*3 = 26.93)	*U* = 456.00, *Z* = −0.12, *p* = 0.908
Testosterone (2D:4D ratio)	*M* = 0.97 ± .03	*M* = 0.95 ± .04	*T*(59) = 1.75, *p* = 0.085
Physical activity (MET)	*Mdn* = 4980.00 (*Q*1 = 2920.00, *Q*3 = 10,300.00)	*Mdn* = 4010.00 (*Q*1 = 2460.00, *Q*3 = 7500.00)	*U* = 371.00, *Z* = −1.34, *p* = 0.179
Crystalline intelligence (MWT-B, *IQ*)	*Mdn* = 104.00 (*Q*1 = 100.00, *Q*3 = 115.00)	*Mdn* = 101.00 (*Q*1 = 97.75, *Q*3 = 112.00)	*U* = 426.00, *Z* = −0.55, *p* = 0.582
Fluid intelligence (LPS-3, *IQ*)	*M* = 114.90 ± 9.19	*M* = 117.47 ± 9.98	*T*(59) = −1.04, *p* = 0.301
Psychiatric symptoms (BPRS total)	*Mdn* = 19.00 (*Q*1 = 18.00, *Q*3 = 20.00)	*Mdn* = 19.00 (*Q*1 = 18.00, *Q*3 = 21.00)	*U* = 390.50, *Z* = −1.11, *p* = .268
Psychopathy (PPI-R total, *T*)	*Mdn* = 55.00 (*Q*1 = 44.00, *Q*3 = 60.00)	*Mdn* = 49.50 (*Q*1 = 43.25, *Q*3 = 55.50)	*U* = 381.50, *Z* = −1.19, *p* = 0.233
Childhood trauma (CTQ total)	*Mdn* = 30.00 (*Q*1 = 25.00, *Q*3 = 36.50)	*Mdn* = 32.50 (*Q*1 = 27.50, *Q*3 = 38.75)	*U* = 391.50, *Z* = −1.05, *p* = 0.293

*Notes.* *BMI* Body-mass-index, *BPRS* brief psychiatric rating scale (CIPS 1977), *CTQ* Childhood Trauma Questionnaire (Bernstein et al. 2003), *EHI* Edinburgh Handedness Inventory (Oldfield 1971), *IQ* intelligence quotient, *LPS-3* performance testing system, subtest: logical thinking (Horn 1983); *Mdn* median, MET metabolic equivalent, *MWT-B* multiple-choice vocabulary test (Lehrl 2005), version B, *n* number, *PPI-R* psychopathic personality inventory-revised (Alpers and Eisenbarth 2008), *Q1*  first quartile, *Q3* third quartile

The martial artists reported having a median martial arts experience of *Mdn* = 11.50 years (*Q*1 = 6.85, *Q*3 = 17.00). *N* = 21 of them had competed in the past (*Mdn* = 6.00 times, *Q*1 = 3.00, *Q*3 = 20.00).

We tested whether the two groups differed in their experiences of violence, aggressiveness, and empathy. Both groups reported comparable personal experiences with violence (*χ*^2^(1) = 2.63, *p* = 0.105). Martial artists showed significantly higher scores on the scales appetitive aggression (AFAS; especially passive and physical aspects of appetitive aggression), *U* = 161.50, *Z* = −4.39, *p* < 0.001, facilitative aggression (AFAS; especially passive and physical aspects of facilitative aggression), *U* = 358.50, *Z* = −2.99, *p* = 0.003, physical aggression (BPAQ), *U* = 147.50, *Z* = −4.59, *p* < 0.001, and aggressive actions (BPAQ), *U* = 180.00, *Z* = −4.11, *p* < 0.001. However, controls had significantly higher scores on the QCAE’s scale for affective empathy, *T*(59) = 2.08,* p* = 0.042. There were no differences for verbal aggression (AFAS, BPAQ), anger (BPAQ), hostility (BPAQ), aggressive thoughts (BPAQ), or cognitive empathy (QCAE). Table 2 provides a detailed overview of the intramural characteristics when comparing martial artists and controls.

**Table 2:** Intramural characteristics of the martial artists compared to controls

	Martial artists (*n* = 29)	Controls (*n* = 32)	Statistics
Victim of violence			
Yes	*n* = 14	*n* = 9	
No	*n* = 15	*n* = 23	*χ*^2^(1) = 2.63, *p* = 0.105
AFAS			
Appetitive aggression	*Mdn* = 8.00 (*Q*1 = 6.00, *Q*3 = 12.50)	*Mdn* = 2.50 (*Q*1 = .25, *Q*3 = 5.75)	*U* = 161.50, *Z* = −4.39, *p* < 0.001^***^
Passive	*Mdn* = 4.00 (*Q*1 = 2.00, *Q*3 = 6.00)	*Mdn* = 1.00 (*Q*1 = .00, *Q*3 = 2.00)	*U* = 227.50, *Z* = −3.46, *p* = 0.001^**^
Verbal	*Mdn* = 1.00 (*Q*1 = .00, *Q*3 = 3.00)	*Mdn* = 0.00 (*Q*1 = .00, *Q*3 = 2.75)	*U* = 370.00, *Z* = −1.42, *p* = 0.156
Physical	*Mdn* = 3.00 (*Q*1 = 1.50, *Q*3 = 5.50)	*Mdn* = 0.00 (*Q*1 = .00, *Q*3 = .00)	*U* = 99.00, *Z* = −5.56, *p* < 0.001^***^
Facilitative aggression	*Mdn* = 8.00 (*Q*1 = 5.00, *Q*3 = 11.00)	*Mdn* = 4.00 (*Q*1 = 3.00, *Q*3 = 7.00)	*U* = 358.50, *Z* = −2.99, *p* = 0.003^**^
Passive	*Mdn* = 3.00 (*Q*1 = 2.00, *Q*3 = 6.00)	*Mdn* = 3.00 (*Q*1 = 1.00, *Q*3 = 4.00)	*U* = 309.50, *Z* = −2.26, *p* = 0.024^*^
Verbal	*Mdn* = 2.00 (*Q*1 = 1.00, *Q*3 = 3.00)	*Mdn* = 2.00 (*Q*1 = .00, *Q*3 = 3.75)	*U* = 443.50, *Z* = −0.31, *p* = 0.757
Physical	*Mdn* = 2.00 (*Q*1 = 1.00, *Q*3 = 3.00)	*Mdn* = 0.00 (*Q*1 = 0.00, *Q*3 = .00)	*U* = 183.00, *Z* = −4.37, *p* < 0.001^***^
BPAQ			
Aggressive actions	*Mdn* = 28.00 (*Q*1 = 25.00, *Q*3 = 32.00)	*Mdn* = 22.50 (*Q*1 = 19.25, *Q*3 = 26.00)	*U* = 180.00,* Z* = −4.11,* p* < 0.001^***^
Physical aggression	*Mdn* = 16.00 (*Q*1 = 14.00, *Q*3 = 21.50)	*Mdn* = 12.00 (*Q*1 = 10.25, *Q*3 = 14.75)	*U* = 147.50, *Z* = −4.59, *p* < 0.001^***^
Verbal aggression	*M* = 11.69 ± 2.52	*M* = 10.38 ± 2.67	*T*(59) = 1.97, *p* = 0.054
Aggressive thoughts/feelings	*Mdn* = 22.00 (*Q*1 = 20.00, *Q*3 = 27.50)	*Mdn* = 23.50 (*Q*1 = 19.25, *Q*3 = 29.00)	*U* = 437.50, *Z* = −0.38, *p* = 0.701
Anger	*Mdn* = 10.00 (*Q*1 = 10.00, *Q*3 = 12.00)	*Mdn* = 10.50 (*Q*1 = 9.00, *Q*3 = 13.00)	*U* = 440.00, *Z* = −0.35, *p* = 0.726
Hostility	*Mdn* = 12.00 (*Q*1 = 10.00, *Q*3 = 15.00)	*Mdn* = 13.00 (*Q*1 = 10.25, *Q*3 = 16.50)	*U* = 407.50, *Z* = −0.82, *p* = 0.412
QCAE			
Affective empathy	*M* = 28.14 ± 4.06	*M* = 30.05 ± 4.75	*T*(59) = −2.08,* p* = 0.042^*^
Cognitive empathy	*M* = 55.90 ± 6.93	*M* = 58.13 ± 7.28	*T*(59) = −1.22, *p* = 0.228

*AFAS* Appetitive and Facilitative Aggression Scale (work in progress, civil version of Appetitive Aggression Scale (AAS; Weierstall and Elbert 2011), *BPAQ* Buss–Perry Aggression Questionnaire (Werner and von Collani 2014), *Mdn* median, *n* number, *QCAE*  Questionnaire of Cognitive and Affective Empathy (Reniers et al. 2011), *Q1* first quartile, *Q3* third quartile

In terms of general morphological brain differences, martial artists (*M*_*TIV*_ = 1576.72 ± 112.40; *M*_*Thickness*_ = 2.74 ± 0.11) and controls (*M*_*TIV*_ = 1573.06 ± 103.81; *M*_*Thickness*_ = 2.75 ± 0.09) did not differ in their TIV, *T*(59) = 0.13, *p* = 0.895, nor in their mean thickness, *T*(59) = 0.43, *p* = 0.668. Furthermore, the median IQRs of both groups [martial artists: *Mdn* = 86.50 (*Q*1 = 86.19, *Q*3 = 86.61); controls: *Mdn* = 86.45 (*Q*1 = 85.58, *Q*3 = 86.61)] were comparable, *U* = 343.00, *Z* = −1.75, *p* = 0.080. Supplementary Table S2 provides detailed information on the brain morphological characteristics of both groups.

### VBM analysis

**Fig. 1:**
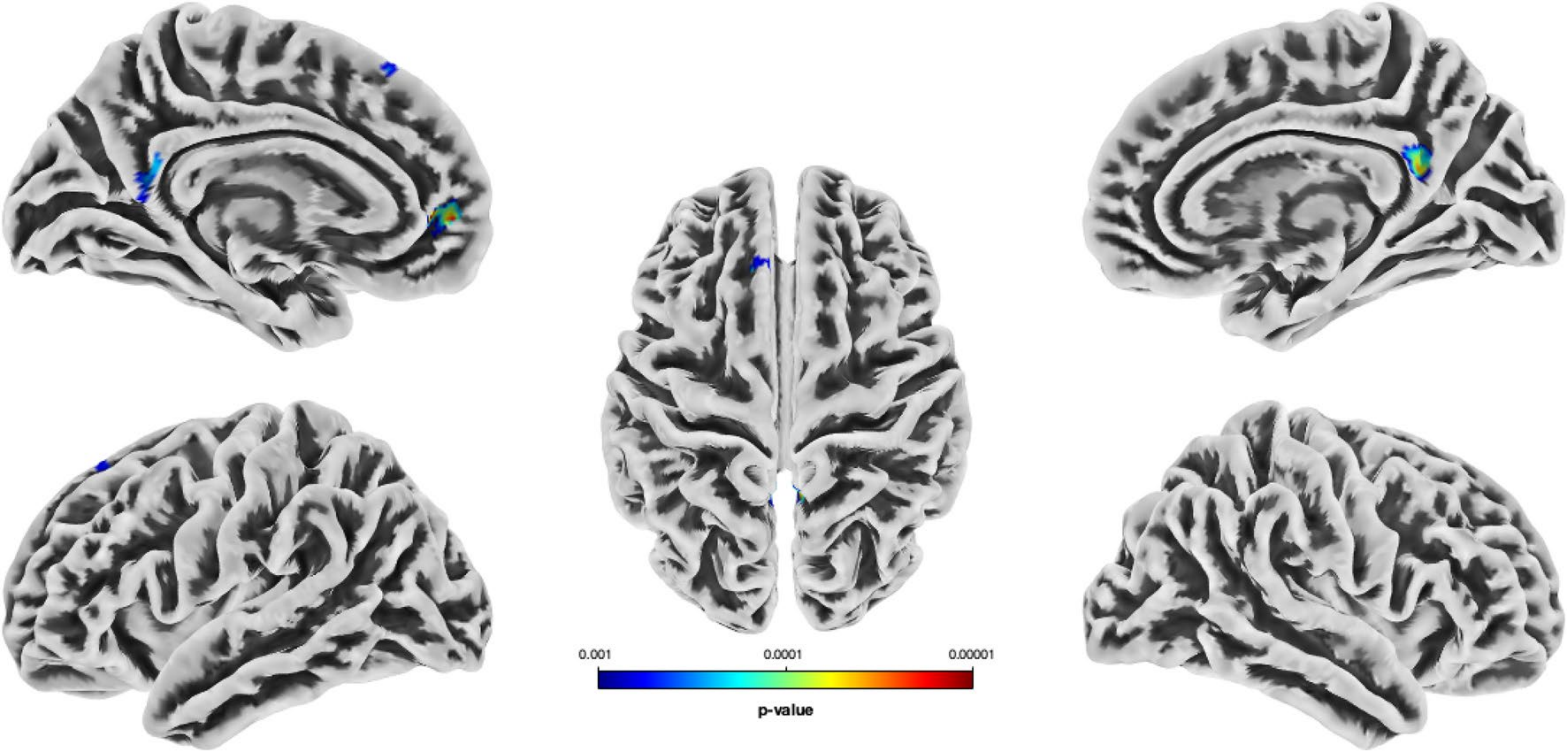
Increased GM volume for martial artists compared to controls in three brain clusters: cluster 1 includes parts of the left superior frontal gyrus, left supplementary motor area and left (para-)cingulate gyri, cluster 2 includes parts of the bilateral superior frontal gyrus and bilateral ACC, and cluster 3 includes parts of the bilateral posterior cingulate gyrus and bilateral precuneus, *t* test, whole brain: *p* < 0.001, cluster level: FWE-corrected (corrected for multiple comparisons). The color bar reflects the *p*-value

We observed differences in regional GM volume between the two groups: martial artists had significantly increased GM volumes in three clusters compared to controls (*t* test, whole brain: *p* < 0.001; see Fig. 1). Cluster 1 includes the left superior frontal gyrus (dorsolateral and medial parts), the left supplementary motor area, and the left middle cingulate and paracingulate gyri (cluster level: *p*_*FWE-corr*_ = 0.022). The second cluster includes the bilateral superior frontal gyrus (medial and medial orbital parts) and the bilateral pregenual ACC (cluster level: *p*_*FWE-corr*_ = 0.013). Cluster 3 comprises the bilateral posterior cingulate gyrus and the bilateral precuneus (cluster level: *p*_*FWE-corr*_ = 0.007). There was no significant effect for the “controls > martial artists” t contrast. Table 3 summarizes the MRI statistics.

**Table 3:** MRI statistics

Anatomical area^a^	*p*_*FWE*_ (cluster level)	*k*_E_	*T*	*p*_*uncorr*_ (peak-level)	Coordinates of peak voxel
Cluster 1	0.022^*^	327	5.03	< 0.001^***^	–12, 27, 48
L superior frontal gyrus, dorsolateral (43.5%)					
L superior frontal gyrus, medial (27.2%)					
L supplementary motor area (25.8%)					
L middle cingulate and paracingulate gyri (1.8%)					
Cluster 2	0.013^*^	362	4.73	< .0001^***^	−4, 52, 0
L superior frontal gyrus, medial (38.4%)					
L anterior cingulate cortex, pregenual (33.3%)					
L superior frontal gyrus, medial orbital (12.0%)					
R superior frontal gyrus, medial (10.0%)					
R superior frontal gyrus, medial orbital (4.6%)					
R anterior cingulate cortex, pregenual (0.2%)					
Cluster 3	0.007^**^	410	4.41	< 0.001^***^	2, −50, 16
L posterior cingulate gyrus (24.8%)					
R precuneus (23.7%)					
R posterior cingulate gyrus (18.6%)					
L precuneus (14.1%)					

Overview of significant clusters for *t* contrast martial artists > controls and dependent variable GM volume

*FWE* family-wise error, *k*_*E*_ = cluster size (voxel), *L* left, *R*  right, *GM*  grey matter

^a^Atlas: AAL3 (Rolls et al. 2020)

**Fig. 2:**
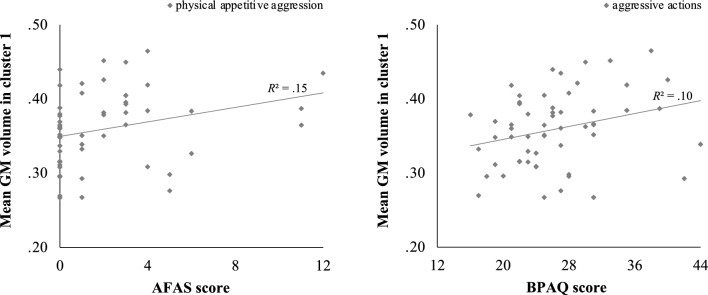
Positive correlation between mean GM volume in cluster 1 (specifically, left superior frontal gyrus and left supplementary motor area) and raw scores on the physical appetitive aggression subscale of the AFAS, *r* = 0.39, *p* = 0.004, and the aggressive actions subscale of the BPAQ, *r* = 0.32, *p* = 0.018

**Fig. 3:**
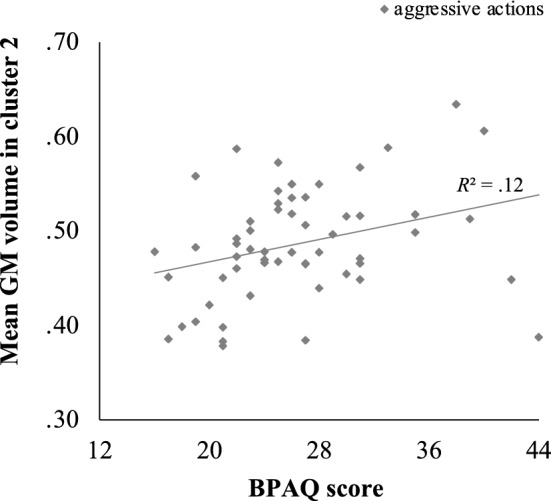
Positive correlation between mean GM volume in cluster 2 (specifically, left superior medial frontal gyrus, bilateral medial frontal cerebrum, and left anterior cingulate gyrus) and raw score on aggressive actions subscale of the BPAQ, *r* = 0.34, *p* = 0.011

**Fig. 4:**
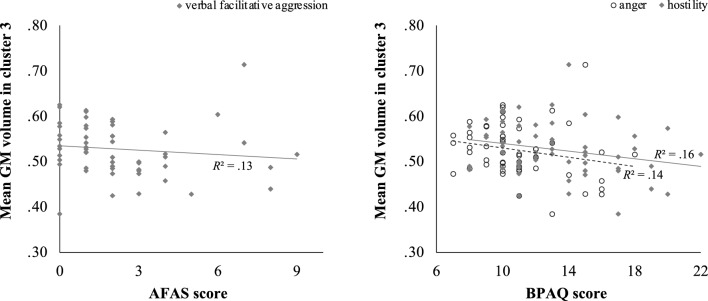
Negative correlation between mean GM volume in cluster 3 (bilateral posterior cingulate gyrus, bilateral precuneus) and raw scores on verbal facilitative aggression subscale of the AFAS, *r* = −0.36, *p* = 0.007, and the BPAQ subscales anger, *r* = −0.38, *p* = 0.004, and hostility, *r* = −0.40, *p* = 0.002

Across all subjects, mean GM volume in cluster 1 was positively associated with physical appetitive aggression (AFAS), *r* = 0.39, *p* = 0.004, and with aggressive actions (BPAQ), *r* = 0.32, *p* = 0.018; see Fig. 2. Mean GM volume in cluster 2 was also positively correlated with the aggressive actions subscale of the BPAQ, *r* = 0.34, *p* = 0.011; see Fig. 3. In addition, GM volume in cluster 3 was negatively correlated with the AFAS subscale verbal facilitative aggression, *r* = −0.36, *p* = 0.007, and the two BPAQ subscales anger, *r* = −0.38, *p* = 0.004, and hostility, *r* = −0.40, *p* = 0.002; see Fig. 4. There were no significant interactions of GM volume in the three clusters with the subscales of the QCAE.

### SBM analysis

**Fig. 5:**
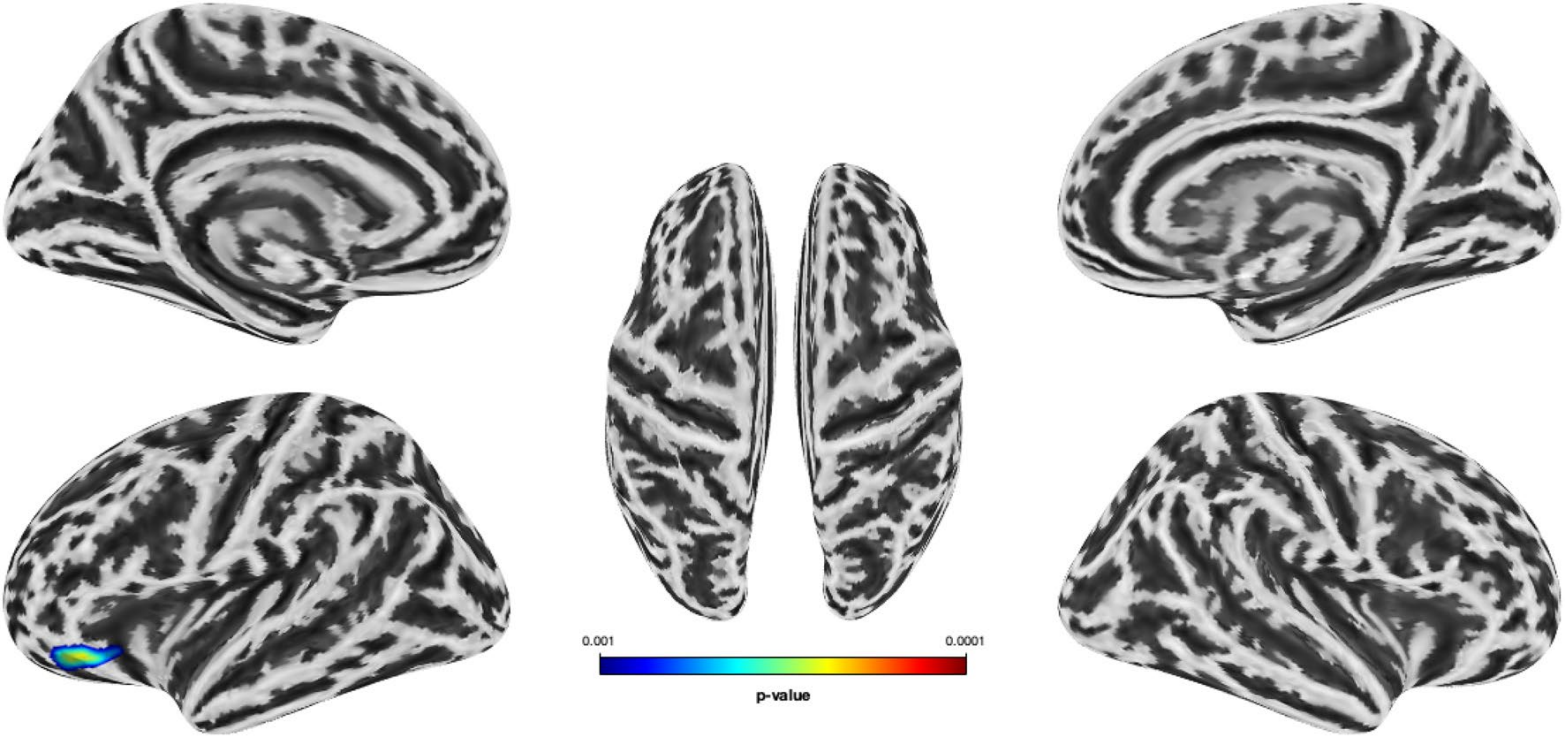
Trend finding of a considerably increased GI in martial artists compared to controls in the left lateral orbital frontal cortex and left pars orbitalis, *t* test, whole brain: *p* < 0.001, cluster level: FWE-corrected (not corrected for multiple comparisons). The underlying surface is the inflated average surface. The color bar reflects the *p*-value

In terms of cortical thickness, we could not find significant differences between martial artists and controls in the second-level analysis. A considerably more pronounced GI was observed in the martial artists in a cluster that included the left lateral orbital frontal cortex and the left pars orbitalis (*t* test, whole brain: *p* < 0.001, cluster level: *p*_*FWE-corr*_ = 0.033). However, this potential effect did not survive the correction for multiple testing. The *t *contrast ‘controls > martial artists’ also showed no significant effects (Fig. 5; Table 4).

**Table 4:** MRI statistics

Overlap of atlas^a^ region	Cluster size	*p*_*FWE*_ (cluster level)	*T*
Cluster 1	198	0.033	3.73
L lateral orbital frontal cortex (62.0%)			
L pars orbitalis (38.0%)			

Summary of trend findings for the *t *contrast martial artists > controls and the dependent variable gyrification index (not corrected for multiple comparisons)

*FW* family-wise error, *L* left

^a^Atlas: Desikan–Killiany DK40 (Desikan et al. 2006)

## Discussion

The aim of the present study was to investigate the relationship between brain morphology and aggressiveness in two healthy samples, one of which was prone to aggressive behavior in the context of its recreational activities (martial arts). Using high-resolution structural MRI, we identified group differences in GM volume in two frontal and one parietal brain clusters, as well as trend findings in GI variations in one frontal brain cluster.

Martial artists showed significantly increased mean GM volumes in two frontal (mainly bilateral superior frontal gyrus, bilateral ACC) and one parietal (mainly bilateral posterior cingulate gyrus, bilateral precuneus) brain clusters compared to control subjects. These anatomical regions are particularly involved in the processing and regulation of emotions, including anger and aggression (Dalwani et al. 2011; Besteher et al. 2017; Bogerts et al. 2017; Zhang et al. 2018) and/or are relevant for theory of mind and overlap with cortical regions relevant for empathic processes (Bernhardt and Singer 2012; Kanske et al. 2015; Schmidt et al. 2017). The superior frontal gyrus, located in the upper part of the PFC, is essential for social cognition, especially perspective taking (Rogers and De Brito 2016). The ACC, also a prefrontal structure, is part of the emotion regulation circuitry (Bogerts et al. 2017). Structural changes in this limbic region have been repeatedly associated with aggressive/antisocial behavior or psychopathic traits (Koenigs 2012; Rosell and Siever 2015; Smith et al. 2016; Raine 2019). The precuneus and the posterior cingulate gyrus are parietal structures that are both part of the default mode network. They are involved in moral/social cognition and empathic aspects of proactive aggression (Westlye et al. 2017; Zhu et al. 2019). In our post hoc analysis, we report positive correlations between GM volumes in the two prefrontal clusters and several aggression subscales, specifically mapping physical/reactive aspects of aggression. Consistent with our findings, Besteher et al. (2017) reported a positive association between GM volumes in the left superior (medial) frontal as well as left anterior cingulate gyrus and aggression in a large healthy cohort. In addition, patients with conduct disorder (CD) had significantly higher GM volumes in the left precuneus, ACC, and superior frontal gyrus as compared to controls (Zhang et al. 2018). Schiffer et al. (2013) found that men with schizophrenia and a history of CD had larger GM volumes in the left cuneus/precuneus and inferior parietal cortex compared to men with schizophrenia without CD. Aoki et al. (2014), in a VBM study of antisocial behavior, reported an increase in GM volume in the right cingulate gyrus but also a decrease in GM volume in the left superior frontal gyrus. Some other studies report conflicting results regarding GM volume/concentration reductions associated with aggressiveness: GM volume reductions in the right superior frontal gyrus have been associated with antisocial behavior (Hofhansel et al. 2020), whereas a negative correlation between GM volume in the left superior medial frontal gyrus and hostility was found in violent patients with schizophrenia (Liu et al. 2020a). For the precuneus/posterior cingulate gyrus area, a reduction in GM volume has been described in subjects with (APD and) psychopathy (Bertsch et al. 2013; Contreras-Rodriguez et al. 2015) and in violent patients with schizophrenia (Kuroki et al. 2017), and a reduction in GM density has been found in male violent offenders with psychopathy (Boccardi et al. 2011).

In terms of gyrification, we report a trend for increased GI levels in martial artists compared to controls in one prefrontal cluster that includes the left lateral orbital frontal cortex and the left pars orbitalis. The pars orbitalis is the orbital division of the inferior frontal gyrus, which has been described to be involved in emotional empathy and mentalizing (Bernhardt and Singer 2012; Buades-Rotger et al. 2017). Consistent with our findings, Storvestre et al. (2019) found increased folding patterns for the left lateral orbitofrontal gyrus in schizophrenic patients with a history of violence compared to those without a history of violence. Furthermore, Schoretsanitis et al. (2019) highlighted the role of GM volume in the left inferior frontal gyri in schizophrenic patients with a history of aggression. However, the GI trend findings did not survive correction for multiple comparisons. In contrast to our strong VBM findings and trend differences in gyrification, we did not find robust effects related to cortical thickness. Although cortical thickness is often correlated with GM measures, there is not a complete overlap (Besteher et al. 2017).

There is ample evidence in the literature for reduced GM volumes in frontal regions, such as the OFC (Gansler et al. 2009; Matsuo et al. 2009), temporal regions, such as the superior temporal cortex (Müller et al. 2008), the (para-)hippocampus (Stevens and Haney-Caron 2012), the temporal pole (Bertsch et al. 2013) and especially the amygdala (Matthies et al. 2012; Pardini et al. 2014), as well as parietal regions (Tiihonen et al. 2008) in individuals with a propensity to violence or aggressive behavior. The lack of significant associations between aggressiveness and GM changes in other associated brain regions, as well as the inverse effects in our study (positive rather than inverse association effects), was not expected. It is conceivable that these contrasting results are due to the fundamental differences between clinical/forensic and community samples. Roberts and colleagues (Roberts et al. 2021) hypothesize that changes in amygdala volume associated with aggression, in particular, are due to comorbid affective or rule-violating symptoms that are not present in a population-based sample. Our study excluded subjects with a psychiatric or neurological disorder and/or psychotropic medical treatment and is therefore not directly comparable with previous studies in clinical samples with severe psychopathology or behavioral disturbances. A critical issue of previous studies is the confounding influence of a comorbid substance use disorder on brain morphology. It is well described that substance use disorder is associated with volumetric GM reductions (Kuroki et al. 2017; Mon et al. 2014), for example, in the OFC and the ventromedial PFC (Schiffer et al. 2011), leading to complications in the interpretation of volumetric changes (Liu et al. 2020). The same is true for the influence of intelligence on the brain structure (Goriounova and Mansvelder 2019). Not all of the above studies reported or controlled for potential confounders, such as age, handedness, IQ, TIV, history of childhood maltreatment, concussion, incarceration/ hospitalization, psychopathology, use of psychotropic medication, or substance use disorders (Pardini et al. 2014; Romero-Martínez et al. 2019). Another methodological difference is that most studies conducted hypothesis-driven region of interest analyses, whereas in the present study, we used a fairly conservative whole-brain analysis approach.

Thus, an important advantage of our nonclinical approach is the absence of some of the above-mentioned confounding variables (Pawliczek et al. 2013). Furthermore, we included only males to avoid gender-specific confounding effects (Dambacher et al. 2015). Using a health questionnaire, we recorded and controlled for a history of psychotropic drug or substance use and concussions. In addition, the groups did not differ with respect to age, IQ, handedness, psychopathic traits, early childhood stress, and smoking. Related to our specific sample of martial artists, there were also no differences in BMI and physical activity between our two groups.

Despite the advantages of including a well-characterized sample and controlling for various confounding factors, there are also some potential limitations that should be considered when interpreting our results. First, our study is based on a cohort of 61 participants. With a larger cohort, we may have been able to detect smaller effects, such as differences in cortical thickness. It should be noted that in our special sample of martial artists, only 30% of the volunteers screened met our strict inclusion criteria (most common exclusion criteria: MRI contraindications, such as tattoos, inappropriate kind of martial arts). The careful selection of participants in hard martial arts (in the sense of combat sports, such as boxing and MMA) was essential for our study paradigm, as previous research has shown significant differences in hostility and aggression between this group and traditional martial arts (Kuśnierz et al. 2014; Kostorz and Sas-Nowosielski 2021). Second, as with most previous MRI studies of aggressive behavior, our cross-sectional approach does not allow us to determine whether the reported structural differences are a cause or a consequence of the observed group differences. Third, aggressiveness in our two groups was measured by self-report questionnaires. This type of instruments can be influenced by subjectivity and social desirability (Vigil-Colet et al. 2012). Nevertheless, the BPAQ in particular is a commonly used instrument in this area of research and, in addition, some of these questionnaires used also provide scales for control/openness. Finally, critical aspects in the specific case of our martial arts sample are increased physical activity and the presence of concussions. Physical exercise and motor skill learning have been associated with changes in the regional brain morphology (Schlaffke et al. 2014), e.g., in athletes, such as dancers (Wei et al. 2011), golfers (Jäncke et al. 2009), or martial artists such as judokas (Jacini et al. 2009). However, we controlled for the physical activities of our two groups and found no group differences. It is well described that cumulative head trauma can lead to structural changes in the brain (Bernick et al. 2015) and is the leading cause of a neurodegenerative disease called chronic traumatic encephalopathy (CTE; Gardner et al. 2014; Tator 2014), which is associated with impulse control problems and aggressiveness, among other neuropsychiatric sequelae (Aaronson et al. 2020). Because participation in contact sports such as martial arts has been associated with CTE (Stern et al. 2011), we collected and controlled for a history of traumatic brain injury using a health questionnaire. In addition, macroscopic neuro-radiological evidence of CTE was assessed by blinded grading by two independent clinically experienced raters. No structural abnormalities were detected, including in brain structures typically associated with repetitive head trauma, e.g., thalamus and caudate (Bernick et al. 2015). In addition, no cognitive impairments or general brain morphological differences were found between the two groups.

Overall, this study shows robust group differences in GM volumes in two community samples that differ only in their propensity for aggressive behavior. Structural brain differences were found in prefrontal (superior frontal gyrus and ACC) and parietal (posterior cingulate gyrus and precuneus) regions, which are important brain areas for the processing and regulation of emotions, such as anger and aggression, as well as empathic processes. Follow-up studies with other and larger community samples prone to aggressive behavior with longitudinal and additional functional measures are planned. In addition, the use of other imaging techniques (e.g., diffusion tensor imaging) will be of interest to determine the interplay between the regions described.

### Supplementary Information

Below is the link to the electronic supplementary material.

Supplementary file1 (PDF 21 KB)

Supplementary file2 (PDF 15 KB)

## Data Availability

The data that support the findings of this study are available from the corresponding author upon reasonable request.
