# Supplementary material for: Neuroanatomical correlates of aggressiveness: a case–control voxel- and surface-based morphometric study
Source: Brain Struct Funct. 2023 Oct 11;229(1):31–46. doi: 10.1007/s00429-023-02715-x (PMC10827843; doi:10.1007/s00429-023-02715-x)
Supplement: Supplementary file 1 — Supplementary file1 (PDF 21 KB) [file 429_2023_2715_MOESM1_ESM.pdf]

Title: **Brain structure of martial artists. A case-control voxel- and surface-based morphometric study**

Journal: Brain Structure and Function

Author names: Stephanie Seidenbecher, Maria Schöne, Jörn Kaufmann, Kolja Schiltz, Bernhard Bogerts, & Thomas Frodl

Corresponding author: Stephanie Seidenbecher; Department of Psychiatry and Psychotherapy, Otto von Guericke University Magdeburg, Magdeburg, Germany; Stephanie.Seidenbecher@med.ovgu.de

Table SI 1 Detailed information on psychiatric symptoms (five subscales of the BPRS), psychopathic traits (eight subscales of the PPR-R) and early childhood traumata (five subscales of the CTQ) in martial artists compared to controls.

	Martial artists (*n* = 29)	Controls (*n* = 32)	Statistics
Psychiatric symptoms (BPRS)			
- Anxiety/depression	*Mdn* = 1.00 (*Q1* = 1.00, *Q3* = 1.00)	*Mdn* = 1.00 (*Q1* = 1.00, *Q3* = 1.25)	*U* = 390.50, *Z* = -1.35, *p* = .178
- Anergia	*Mdn* = 1.00 (*Q1* = 1.00, *Q3* = 1.13)	*Mdn* = 1.00 (*Q1* = 1.00, *Q3* = 1.00)	*U* = 436.50, *Z* = -.55, *p* = .579
- Thought disorder	*Mdn* = 1.00 (*Q1* = 1.00, *Q3* = 1.00)	*Mdn* = 1.00 (*Q1* = 1.00, *Q3* = 1.00)	*U* = 443.50, *Z* = -.43, *p* = .669
- Activation	*Mdn* = 1.00 (*Q1* = 1.00, *Q3* = 1.00)	*Mdn* = 1.00 (*Q1* = 1.00, *Q3* = 1.25)	*U* = 378.00, *Z* = -1.93, *p* = .053
- Hostility/mistrust	*Mdn* = 1.00 (*Q1* = 1.00, *Q3* = 1.00)	*Mdn* = 1.00 (*Q1* = 1.00, *Q3* = 1.00)	*U* = 452.00, *Z* = -.46, *p* = .644
Psychopathy (PPI-R)			
- Debt externalization	*Mdn* = 22.00 (*Q1* = 19.50, *Q3* = 27.50)	*Mdn* = 24.50 (*Q1* = 19.25, *Q3* = 30.25)	*U* = 415.50, *Z* = -.70, *p* = .483
- Rebellious risk-taking	*Mdn* = 57.00 (*Q1* = 52.00, *Q3* = 64.50)	*Mdn* = 52.00 (*Q1* = 42.25, *Q3* = 59.75)	*U* = 317.00, *Z* = -2.13, *p* = .034*
- Stressimmunity	*Mdn* = 50.00 (*Q1* = 43.00, *Q3* = 53.50)	*Mdn* = 45.00 (*Q1* = 41.25, *Q3* = 48.00)	*U* = 339.00, *Z* = -1.82, *p* = .068
- Social influence	*Mdn* = 46.00 (*Q1* = 42.00, *Q3* = 50.50)	*Mdn* = 43.00 (*Q1* = 39.25, *Q3* = 49.75)	*U* = 392.50, *Z* = -1.03, *p* = .301
- Coldheartedness	*Mdn* = 33.00 (*Q1* = 30.00, *Q3* = 38.00)	*Mdn* = 32.50 (*Q1* = 28.25, *Q3* = 39.25)	*U* = 453.50, *Z* = -.15, *p* = .879
- Machiavellian Egoism	*Mdn* = 36.00 (*Q1* = 31.00, *Q3* = 39.00)	*Mdn* = 36.00 (*Q1* = 33.00, *Q3* = 38.00)	*U* = 422.00, *Z* = -.61, *p* = .543
- Careless planlessness	*Mdn* = 30.00 (*Q1* = 25.50, *Q3* = 33.50)	*Mdn* = 27.00 (*Q1* = 26.00, *Q3* = 32.75)	*U* = 416.50, *Z* = -.69, *p* = .492
- Fearlessness	*Mdn* = 20.00 (*Q1* = 18.50, *Q3* = 23.50)	*Mdn* = 20.00 (*Q1* = 14.25, *Q3* = 22.00)	*U* = 368.00, *Z* = -1.39, *p* = .164
Childhood Trauma (CTQ)			
- Emotional abuse	*Mdn* = 5.00 (*Q1* = 5.00, *Q3* = 7.00)	*Mdn* = 6.50 (*Q1* = 5.00, *Q3* = 7.75)	*U* = 382.00, *Z* = -1.26, *p* = .208
- Physical mistreatment	*Mdn* = 5.00 (*Q1* = 5.00, *Q3* = 6.50)	*Mdn* = 5.00 (*Q1* = 5.00, *Q3* = 5.00)	*U* = 376.00, *Z* = -1.78, *p* = .076
- Sexual abuse	*Mdn* = 5.00 (*Q1* = 5.00, *Q3* = 5.00)	*Mdn* = 5.00 (*Q1* = 5.00, *Q3* = 5.00)	*U* = 432.00, *Z* = -1.50, *p* = .134
- Emotional neglect	*Mdn* = 8.00 (*Q1* = 5.00, *Q3* = 12.00)	*Mdn* = 10.00 (*Q1* = 6.25, *Q3* = 13.00)	*U* = 355.50, *Z* = -1.58, *p* = .113
- Physical neglect	*Mdn* = 5.00 (*Q1* = 5.00, *Q3* = 7.00)	*Mdn* = 5.00 (*Q1* = 5.00, *Q3* = 7.00)	*U* = 440.50, *Z* = -.37, *p* = .710

Abbreviations. BPRS = Brief Psychiatric Rating Scale (CIPS 1977); CTQ = Childhood Trauma Questionnaire (Bernstein et al. 2003); PPI-R = Psychopathic Personality Inventory-Revised (Alpers and Eisenbarth 2008).
