# Supplementary material for: Neuroanatomical correlates of aggressiveness: a case–control voxel- and surface-based morphometric study
Source: Brain Struct Funct. 2023 Oct 11;229(1):31–46. doi: 10.1007/s00429-023-02715-x (PMC10827843; doi:10.1007/s00429-023-02715-x)
Supplement: Supplementary file 2 — Supplementary file2 (PDF 15 KB) [file 429_2023_2715_MOESM2_ESM.pdf]

Title: **Brain structure of martial artists. A case-control voxel- and surface-based morphometric study**

Journal: Brain Structure and Function

Author names: Stephanie Seidenbecher, Maria Schöne, Jörn Kaufmann, Kolja Schiltz, Bernhard Bogerts, & Thomas Frodl

Corresponding author: Stephanie Seidenbecher; Department of Psychiatry and Psychotherapy, Otto von Guericke University Magdeburg, Magdeburg, Germany; Stephanie.Seidenbecher@med.ovgu.de

Table SI 2 Morphological brain characteristics of martial artists compared to controls.

	Martial artists (n = 29)	Controls (n = 32)	Statistics
Brain volume estimates [cm^3]			
- CSF	*Mdn* = 260.00 (*Q1* = 235.50, *Q3* = 304.00)	*Mdn* = 286.00 (*Q1* = 250.50, *Q3* = 325.75)	U = 343.50, Z = -1.74, p = .082
- GM	M = 732.59 ± 58.11	M = 710.19 ± 54.92	T(59) = 1.55, p = .127
- WM	M = 568.34 ± 54.78	M = 567.92 ± 44.08	T(59) = .05, p = .963
- TIV	M = 1576.72 ± 112.40	M = 1573.06 ± 103.81	T(59) = .13, p = .895
Mean thickness [mm]	M = 2.74 ± .11	M = 2.75 ± .09	T(59) = -.43, p = .668
Image and Preprocessing Quality			
- IQR [%]	*Mdn* = 86.50 (*Q1* = 86.44, *Q3* = 86.53)	*Mdn* = 86.47 (*Q1* = 86.37, *Q3* = 86.51)	U = 343.00, Z = -1.75, p = .080

Abbreviations. CSF = Cerebrospinal fluid; GM = Gray matter; IQR = Image quality rating (weighted average); TIV = Total intracranial volume; WM = White matter.
